# Functional characterization and identification of mouse *Rad51d *splice variants

**DOI:** 10.1186/1471-2199-10-27

**Published:** 2009-03-27

**Authors:** Aaron M Gruver, Brian D Yard, Campbell McInnes, Changanamkandath Rajesh, Douglas L Pittman

**Affiliations:** 1 Department of Pharmaceutical and Biomedical Sciences, South Carolina College of Pharmacy, University of South Carolina Campus, Columbia, SC 29208, USA; 2 Pathology and Laboratory Medicine Institute, Cleveland Clinic, Cleveland, OH 44195, USA

## Abstract

**Background:**

The homologous recombination (HR) pathway is vital for maintaining genomic integrity through the restoration of double-stranded breaks and interstrand crosslinks. The RAD51 paralogs (RAD51B, RAD51C, RAD51D, XRCC2, XRCC3) are essential for this process in vertebrates, and the RAD51D paralog is unique in that it participates in both HR repair and telomere maintenance. RAD51D is also known to directly interact with the RAD51C and XRCC2 proteins. *Rad51d *splice variants have been reported in mouse and human tissues, supportive of a role for alternative splicing in HR regulation. The present study evaluated the interaction of the *Rad51d *splice isoform products with RAD51C and XRCC2 and their expression patterns.

**Results:**

Yeast-2-hybrid analysis was used to determine that the *Mus musculus Rad51d *splice variant product RAD51DΔ7b (deleted for residues 219 through 223) was capable of interacting with both RAD51C and XRCC2 and that RAD51D+int3 interacted with XRCC2. In addition, the linker region (residues 54 through 77) of RAD51D was identified as a region that potentially mediates binding with XRCC2. Cellular localization, detected by EGFP fusion proteins, demonstrated that each of the splice variant products tested was distributed throughout the cell similar to the full-length protein. However, none of the splice variants were capable of restoring resistance of *Rad51d*-deficient cell lines to mitomycin C. RT-PCR expression analysis revealed that *Rad51dΔ3 *(deleted for exon 3) and *Rad51dΔ5 *(deleted for exon 5)transcripts display tissue specific expression patterns with *Rad51dΔ3 *being detected in each tissue except ovary and *Rad51dΔ5 *not detected in mammary gland and testis. These expression studies also led to the identification of two additional *Rad51d *ubiquitously expressed transcripts, one deleted for both exon 9 and 10 and one deleted for only exon 10.

**Conclusion:**

These results suggest *Rad51d *alternative splice variants potentially modulate mechanisms of HR by sequestering either RAD51C or XRCC2.

## Background

Homologous recombination (HR) is responsible for repairing damage affecting both DNA strands and maintaining chromosome stability [1,2]. In mammals, HR requires the RAD51 family of proteins including RAD51 and the RAD51 paralogs (RAD51B, RAD51C, RAD51D, XRCC2, XRCC3) [3]. Genetic studies have demonstrated that RAD51 family members have non-redundant functions, as individual disruption of each gene confers increased sensitivity to DNA damaging agents and a genome instability phenotype [4-8]. In addition, the paralog proteins interact to form at least two stable complexes: a dimer consisting of RAD51C-XRCC3 and a larger "BCDX2" complex consisting of RAD51B, RAD51C, RAD51D, and XRCC2 [9,10]. RAD51D is unique among the RAD51 family in that it is the only paralog currently known to support telomere maintenance in addition to the DNA repair functions [11].

Alternative pre-mRNA splicing is a mechanism responsible for proteome diversity and gene regulation in higher eukaryotes [12-14]. Splice variants of the *Rad51d *gene have been reported previously in mouse and human tissues, as well as in cancer derived cell lines [15-17]. Similarly, *Rad51d *alternative splice variants have also been identified in *Arabidopsis *[18]. For *Mus musculus*, seven alternative transcripts were identified that are predicted to encode six distinct putative protein isoforms. Alternatively spliced translation products commonly display different or antagonistic biological functions compared to their full-length counterparts [19]. Therefore, changes in the pattern of alternative splicing of regulatory genes could have an impact on physiology and pathogenesis, particularly tumor development and progression [20]. Splice variants of DNA repair genes potentially have the capability to regulate HR. It has been demonstrated that two splice variants of RAD52 increase the frequency of direct-repeat recombination from the same chromatid when expressed in either mammalian cells or yeast [21,22]. Moreover, mutations in the *BRCA1 *and *BRCA2 *genes, known to predispose carriers to breast and ovarian cancers, were found to disrupt exonic splicing enhancers and result in aberrant RNA splicing [23]. Recently, a RAD51 splice variant was uncovered that demonstrated homologous pairing activity similar to that of the full length RAD51 protein [24]. Here, we report the *Mus musculus Rad51d *alternative transcripts encode predicted proteins capable of making specific interactions with RAD51C and XRCC2 and the identification of two novel, ubiquitously expressed *Mus musculus Rad51d *alternative transcripts.

## Results

### Alternative transcripts of Rad51d

**Figure 1:**
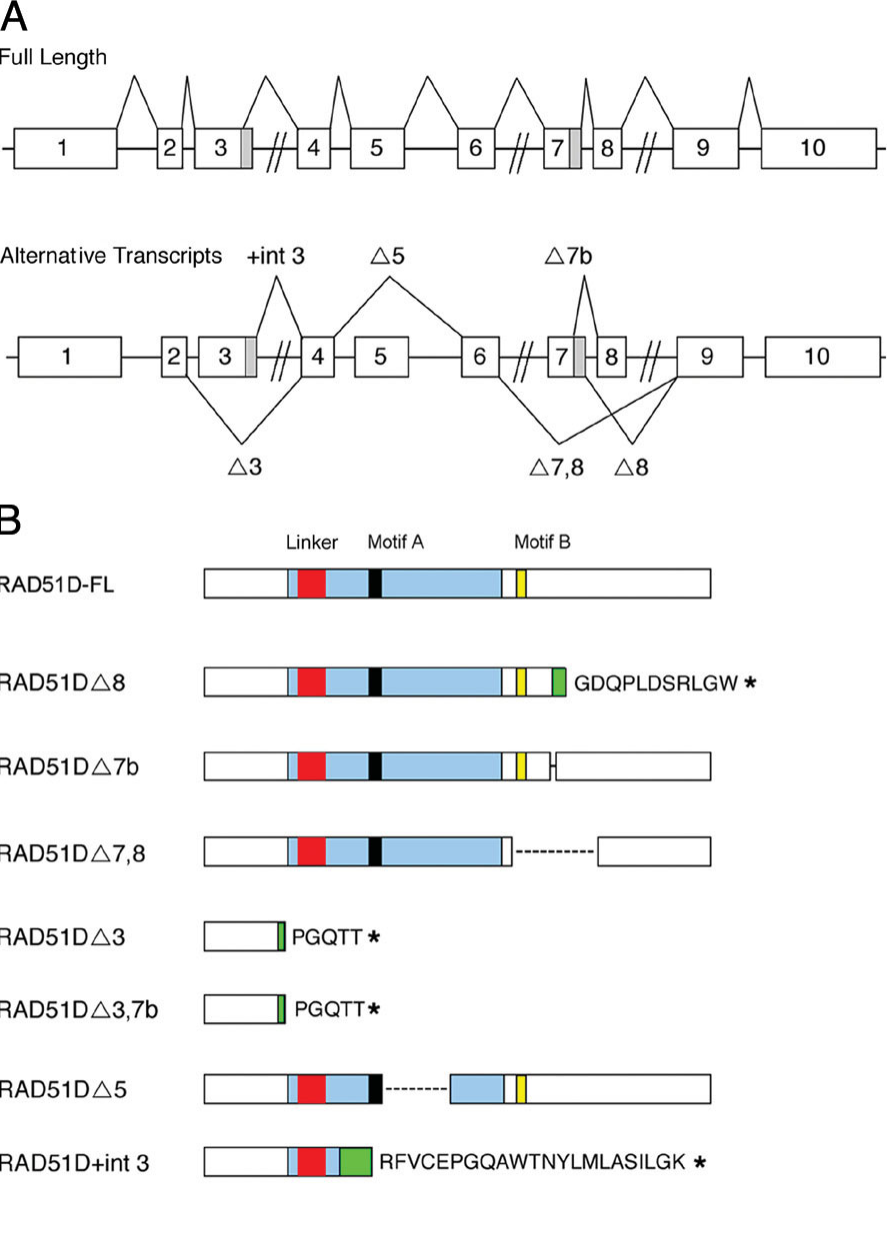
**Summary of alternatively spliced transcripts of *Mus musculus Rad51d***. (A) The ten exons of *Rad51d *are shown as numbered boxes drawn relative to base pair length with both the full-length transcript (upper panel) and each alternatively spliced transcript shown (lower panel). Shaded areas designate partial intron retention (+int3) or partial exon exclusion (Δ7b). (B) Predicted translation products of the RAD51D isoforms are illustrated. Black and yellow boxes indicate the location of Walker Motifs A and B respectively. A core helix-hairpin-helix-GP rich domain (blue) and linker region (red) are also indicated. Amino acids introduced by splicing induced frameshift in the transcripts of RAD51DΔ8, RAD51DΔ3, and RAD51DΔ3,7b are colored green with the corresponding novel sequence following. Asterisks represent sites of premature termination codons.

Multiple *Rad51d *transcripts were first detected by Northern blot analysis [25], and seven splice variants were later identified by RT-PCR in both mouse and human brain tissues [15]. The *Rad51d *gene consists of 10 exons, and a summary of the current evidence for each alternative transcript for the human and mouse *Rad51d *gene from the ASD and EASED databases is presented in Table 1[17,26]. The *Mus musculus Rad51d *alternative transcripts are summarized in Figure 1A and for clarity are referred to as RAD51DΔ (exon excluded) or RAD51D+(intron included). The highly conserved ATP binding Walker Motifs A and B, present in all members of the RAD51 family, are contained within exons 4 and 7 of *Rad51d *respectively (Figure 1B). RAD51D full length (FL) includes both exons 7a and 7b in contrast to the RAD51DΔ7b alternative transcript in which the final 15 base pairs of exon 7 are excluded. Previously, this 3' portion of exon 7 as well as the retained intron in RAD51D+int3 were labeled as additional exons [15]. Internal deletions are also predicted in RAD51DΔ7,8 and RAD51DΔ5 (residues 193–246, and 116–159 respectively), while stretches of novel amino acid sequence and premature stop codons are predicted for the RAD51DΔ8, RAD51DΔ3 and RAD51D+int3 isoforms as a result of splicing induced frameshift mutations (residues 224–233, 49–53, and 88–109 respectively). The splice variants RAD51DΔ3 and RAD51DΔ3,7b are predicted to encode identical peptides.

**Table 1:** Alternative transcripts of the *RAD51D *gene

Variant Name	Representative EST/mRNA	Expression
*HsRAD51DΔ3*	AB016223, DA630921, DA558919, DN997215, BX443779, BI823883, AL597240, NM133627	Brain, kidney, chondrocytes, mammary gland (cancerous), T Cell (Jurkat), lung, testis
*HsRAD51DΔ3,5*	AB016224, DA493777, NM_133630	Brain
*HsRAD51DΔ3,4,5*	AB016225, CD387861, DA422600, DA862519, DB132257, DR005326, N57184, NM133629	Brain, trophoblasts, cervix, placenta, thymus, prostate
*HsRAD51DΔ5*	AB018360	Brain
*HsRAD51DΔ4,5*	AB018361	Brain
*HsRAD51D+int3Δ4,5*	AB018362	Brain
*HsRAD51D+int3*	AB018363, BI915277, DN999128, DC391646	Brain, bone marrow, spleen

		

*MmRAD51DΔ8*	AB052828, BB864057	Brain, bone marrow
*MmRAD51DΔ7b*	AB052829	Brain
*MmRAD51DΔ7,8*	AB052830	Brain
*MmRAD51DΔ3*	AB052831, BB629106	Brain
*MmRAD51DΔ3,7b*	AB052832	Brain
*MmRAD51DΔ5*	AB052833	Brain
*MmRAD51D+int3*	AB052834	Brain

### RAD51D isoforms interact with members of the BCDX2 complex

**Figure 2:**
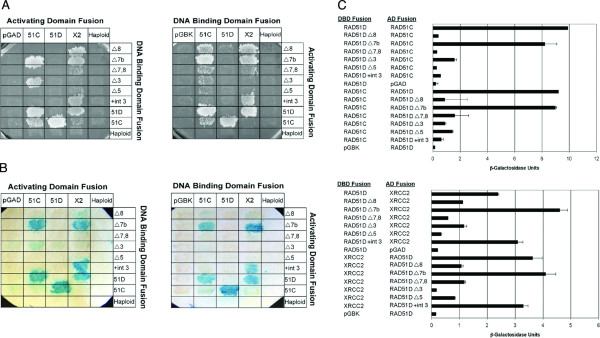
**Interaction of the RAD51D alternative isoforms with RAD51C and XRCC2**. (A) Sixty-four yeast two-hybrid interactions were tested by plating diploid strains on selective growth medium lacking adenine, leucine, histidine, and tryptophan. (B) Colony lift assays were performed to qualitatively assess β-galactosidase activity in mated yeast. (C) Interactions between isoforms of RAD51D with RAD51C and XRCC2 were quantified by measuring β-galactosidase activity using *o*-nitrophenyl-β-D-galactopyranoside as a substrate. The binding capacity of the splice variants with RAD51C and XRCC2 are displayed. Experiments were performed in triplicate with error bars representing standard error of the mean. Abbreviations: *51C*; RAD51C, *51D*; RAD51D-FL, *X2*; XRCC2, *pGAD*; pGADT7 vector, *pGBK*; pGBKT7 vector, *AD*; activating domain of GAL4, *DBD*; DNA binding domain of GAL4.

To investigate whether the predicted RAD51D isoforms interact with binding partners of the full-length protein, each was examined for interaction with RAD51C and XRCC2 by yeast two-hybrid analyses [27]. Yeast expressing the mouse RAD51D-RAD51C and RAD51D-XRCC2 binding partners display growth on selective medium indicating strong protein interactions. Replica plating results suggest that RAD51C interacts with RAD51DΔ7b while XRCC2 interacts with RAD51DΔ8, RAD51DΔ7b and RAD51D+int3 (Figure 2A). The activating domain (AD) fusion of RAD51C suggested interaction with RAD51DΔ3. Mouse RAD51C-RAD51C displayed positive growth when tested in both orientations of the GAL4 fusion, which was not reported with the human RAD51C protein [27,28].

Interaction between RAD51C, XRCC2 and the alternatively spliced isoforms of RAD51D was further assessed by measuring the activity of β-galactosidase. Colony lift assays were performed as a qualitative indicator of enzyme activity. As illustrated in Figure 2B, each of the matings that displayed growth on quadruple dropout medium were positive, with the exception of RAD51C-RAD51C. β-galactosidase activity was weakest for the interactions of RAD51DΔ8 and RAD51DΔ3, suggesting a weaker or transient interaction with RAD51C and XRCC2. To quantify the degree of binding between RAD51D isoforms and RAD51C/XRCC2, β-galactosidase was measured using *o*-nitrophenyl-β-D-galactopyranoside as a substrate (Figure 2C). RAD51DΔ7b displays a level of interaction with RAD51C nearly identical to that of full-length RAD51D. In agreement with colony lift assays however, binding between RAD51DΔ3 and RAD51C was suggested in only one orientation. RAD51DΔ7b and RAD51D+int3 associated with XRCC2 with an affinity similar to the full-length protein. The RAD51DΔ8, RAD51DΔ7,8, and RAD51DΔ3 isoforms display an ability to interact with XRCC2 (36%, 29%, and 22% the level of full-length RAD51D respectively), but variation in these interactions is observed depending upon the orientation of the GAL4 fusion.

### Domain mapping of RAD51D

**Figure 3:**
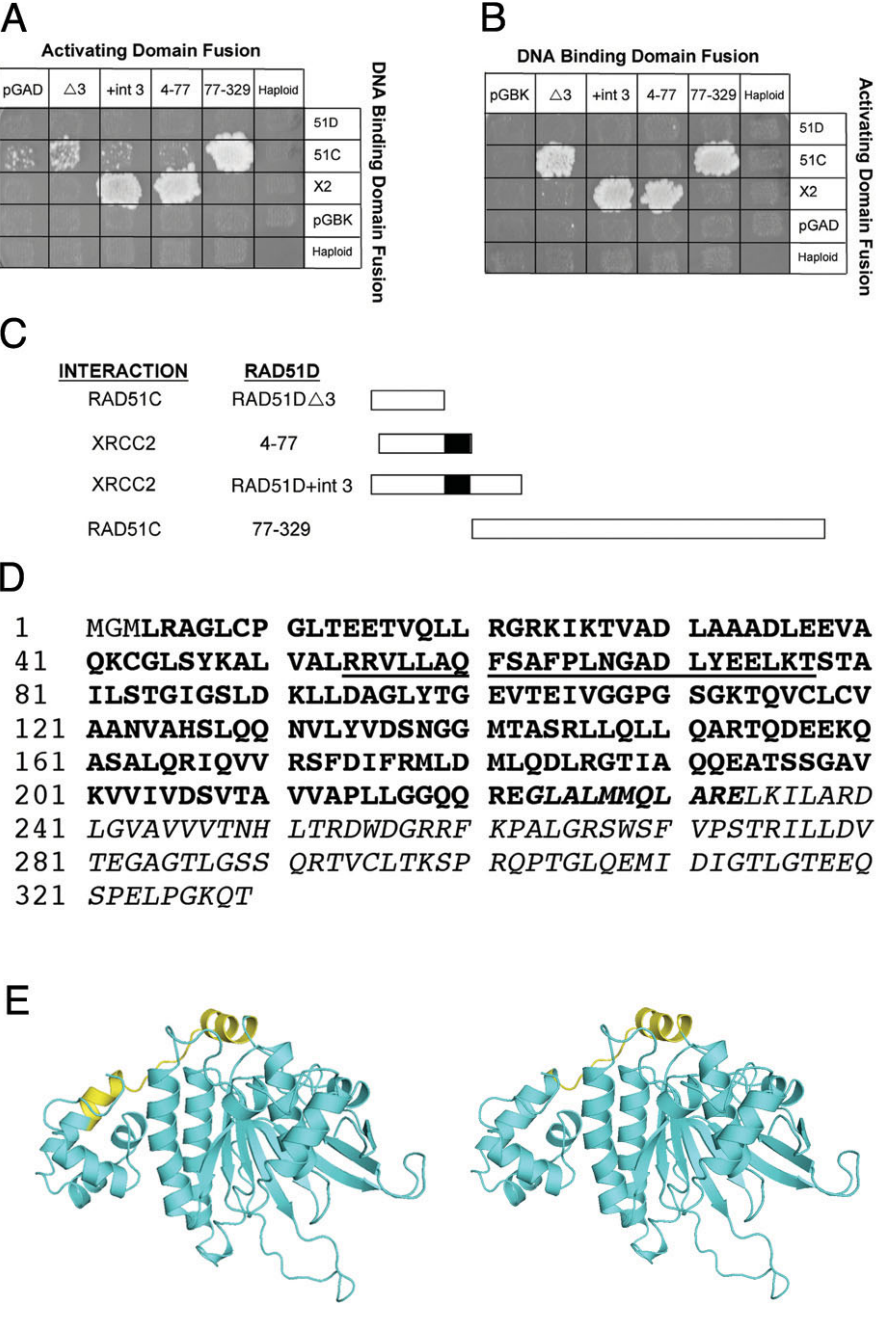
**Domain mapping of *Mus musculus *RAD51D**. (A-B) Yeast two-hybrid analysis of RAD51DΔ3 and RAD51D+int3 are directly compared with the original deletion constructs of RAD51D. (C) Diagram of the RAD51DΔ3 and RAD5D+int3 alternative splice constructs compared with the amino (4–77) and carboxy-terminal (77–329) regions of RAD51D. The black box represents the predicted linker region of RAD51D. (D) The region of the protein that allows interaction with XRCC2 is illustrated in bold type, while sequence required for its interaction with RAD51C is presented in italics (note a small area of overlap between residues 223–233). The underlined 24 amino acid region (residues 54–77) appears to be critical for determining the specificity of the interaction between RAD51D isoforms and XRCC2. (E) Homology model of RAD51D from the *Pyrococcus furiosus *RAD51 crystal structure. The yellow highlighted region (left) represents the linker region and the yellow area (right) represents the 24 amino acid region proposed to determine XRCC2 specificity. Abbreviations: *51D*; RAD51D-FL, *51C*; RAD51C, *X2*; XRCC2, *4–77*; residues 4–77 of the amino-terminal domain of RAD51D, *77–329*; residues 77–329 of the carboxy-terminal domain of RAD51D, *pGAD*; pGADT7 vector, *pGBK*; pGBKT7 vector.

The observation that RAD51D isoforms may selectively interact with RAD51C and XRCC2 allowed further domain mapping of RAD51 paralog complexes. Miller *et al. *reported that the amino-terminal domain of RAD51D (residues 4–77) interacts with XRCC2, and the carboxy-terminal region (residues 77–328) is sufficient for interaction with RAD51C [29]. Binding of RAD51DΔ8 (residues 4–233) and RAD51D+int3 (residues 4–109) to XRCC2 support this observation (Figure 2). However, the lack of association between RAD51DΔ8 and RAD51C suggests a more narrow region of the carboxy-terminal domain than previously reported, consisting of amino acids 234–329, is required for the interaction between RAD51D and RAD51C. Interestingly, the yeast two-hybrid analyses displayed in Figure 2 also suggest that residues 54–77 within the amino-terminal region determine whether RAD51D interacts with XRCC2 (shown in black, Figure 3C). To confirm these observations, the original RAD51D deletion constructs used in the study by Miller *et al *were tested against the RAD51D isoforms. If amino acids 54–77 are present, as with the RAD51D (4–77) construct, the peptide interacts with XRCC2 (Figure 3). When these residues are missing, as in the RAD51DΔ3 isoform, there is an absence of association with XRCC2 and some interaction with RAD51C. This span of 24 amino acids (54–77) is nearly identical to the "linker region" (residues 60–78) proposed from the structure modeling of human RAD51D from the *Pyrococcus furiosus *RAD51 crystal structure [29].

To further illustrate the position of the proposed region responsible for regulating these interactions, a protein homology model was constructed (DiscoveryStudio 2.0, Accelrys). RAD51D was matched to the RAD51 (RADA) structure from *Pyrococcus furiosus *(1PZN) and was shown to have 24% identity and 46% similarity. While the identity is somewhat low, the structural conservation of the RAD51 family supports that meaningful models can be obtained. After construction and refinement, the linker region is similar to what was proposed initially, but according to this model extends further into the alpha-helices of the N-terminal domain (Figure 3E). The overlapping region that potentially regulates RAD51D interaction with XRCC2 and RAD51C is located at the junction between the two domains previously demonstrated to interact with XRCC2 and RAD51C respectively.

### Localization of RAD51D isoforms

RAD51D appears to be present throughout the cell although localization specific to telomeric regions in both meiotic and somatic cells has been demonstrated [11]. To determine if the multiple isoforms of RAD51D display similar localization, amino-terminal enhanced green fluorescent protein (EGFP) tagged constructs were generated and transiently expressed in *Rad51d*-deficient mouse embryonic fibroblasts (MEFs) (see Additional file 1, Panel A). Fluorescent microscopy reveals EGFP-RAD51D-FL is present in both the cytoplasm and nuclear compartments, similar to EGFP vector control. The RAD51D alternatively spliced isoforms also display a mixed cytoplasmic and nuclear distribution within the cell. In contrast, full length EGFP-RAD51C is predominantly nuclear in agreement with the presence of a non-canonical nuclear localization signal (NLS) in its carboxy-terminal region [30,31]. Full-length EGFP-RAD51D was tested for the ability to repair mitomycin C (MMC) induced DNA damage in *Rad51d*-deficient MEFs. The percentage resistance was nearly identical to that observed from cells transfected with RAD51D-FL (see Additional file, Panel B), suggesting the amino-terminal EGFP fusion results in a biologically active protein. Additionally, no change in the localization of EGFP-RAD51D-FL was observed when expressed in wild-type MEFs. This confirms that failure to observe nuclear localization is not due to accumulated mutations in *Rad51d*-deficient MEFs (data not shown).

### Complementation analysis of RAD51D isoforms

**Figure 4:**
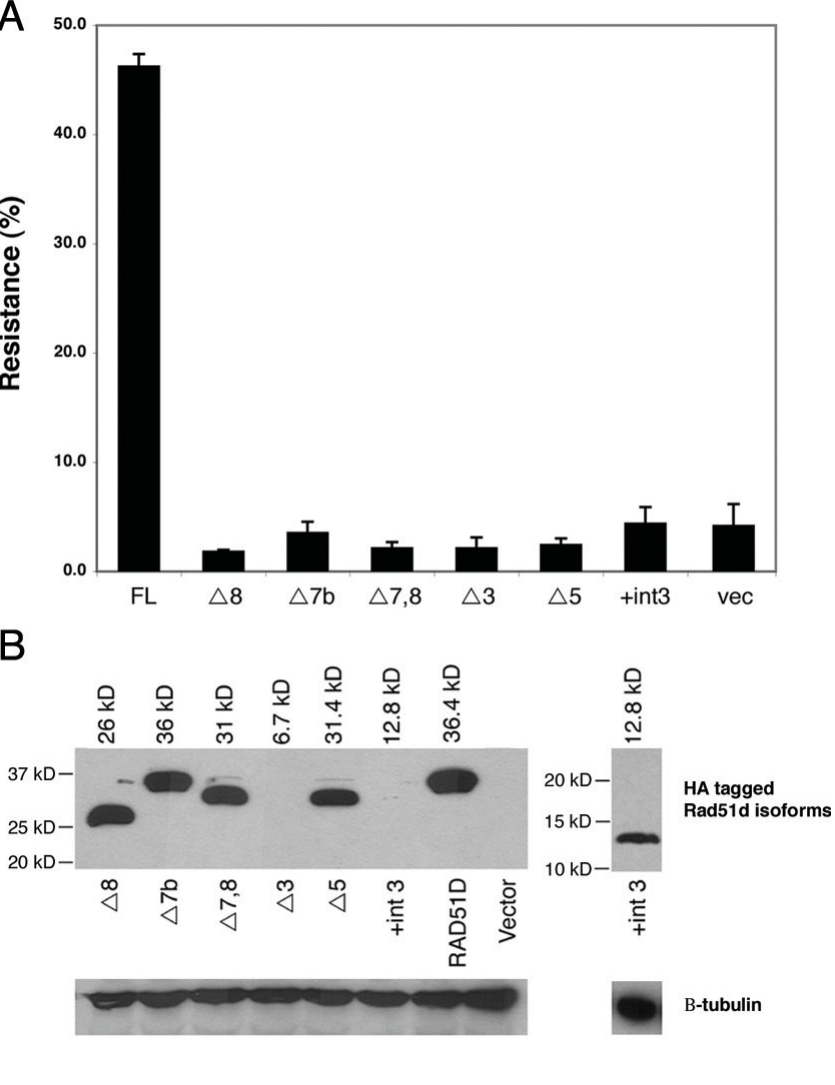
**The putative translation products from alternative splicing of *Mus musculus Rad51d *fail to complement a *Rad51d *deletion**. (A) Alternative transcripts of *Rad51d *were expressed in *Rad51d*^-/-^*Trp53*^-/- ^MEFs. The percentage resistance indicates the fraction of transfectants that survived treatment with 4 ng/mL mitomycin C (MMC). Cells transfected with empty vector (vec) were included as controls. Error bars represent the standard error. (B) Western blot of HA-tagged proteins expressed in *Rad51d*^-/-^*Trp53*^-/- ^MEFs 24 hr post-transfection (upper panel). The expected molecular mass of each isoform is shown above each. Immunoblotting for β-tubulin was performed as a loading control (lower panel). Expression of RAD51DΔ3 could not be verified likely due to the relatively small molecular weight of the putative translation product (6.7 kDa).

Expression of RAD51D-FL was previously demonstrated to correct the DNA interstrand crosslink repair deficiency of *Rad51d*-deficientMEFs [6,32]. To determine whether RAD51D alternative splice products retain any ability to restore cellular resistance to DNA interstrand crosslinking damage caused by exposure to MMC, each was tested in a complementation assay [32]. In the present study, approximately 45% of the population expressing full-length RAD51D was capable of resisting MMC challenge (Figure 4A). However, none of the alternative RAD51D isoforms restored resistance to DNA interstrand cross-links when compared with vector control populations.

### Expression and identification of novel Rad51d alternative transcripts

**Figure 5:**
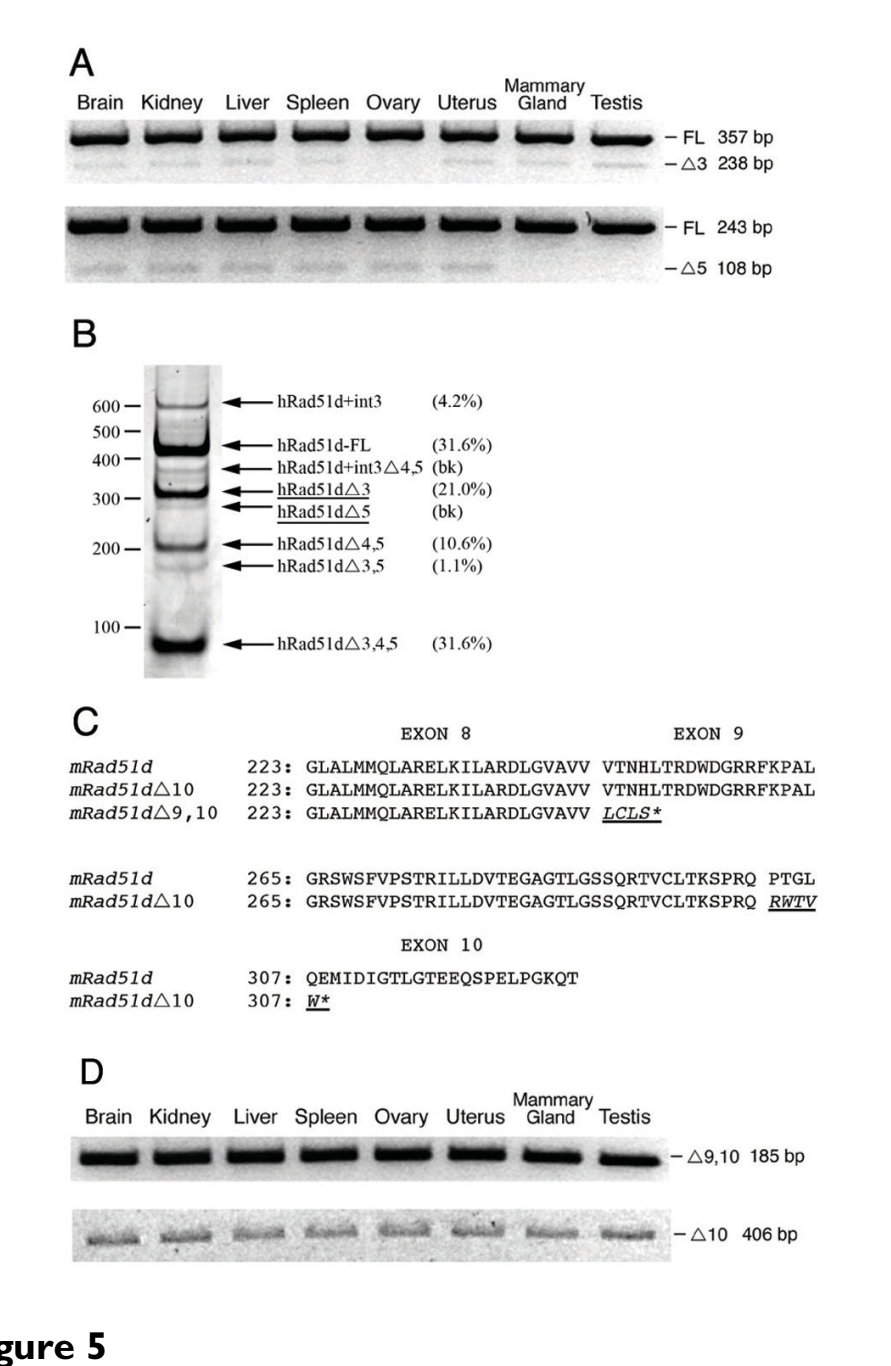
**Expression analysis of *Rad51d *alternative transcripts**. (A) RT-PCR for *Mus musculus Rad51d *and selected splice variants was conducted in eight tissues. Expression of RAD51DΔ3 employing primers Rad51d F1 and R1 (upper panel); RAD51DΔ5 with Rad51d F2 and R2 (lower panel). (B) RT-PCR products of human *RAD51D *in normal breast. Arrows indicate transcripts corresponding to known human splice isoforms [16]. RAD51DΔ3 and RAD51DΔ5 transcript positions are underlined. Size markers in base pairs are shown on the left. Numbers in parenthesis represent transcript abundance as determined by ImageJ analysis of the band intensities (*bk*; background). (C) The RAD51D peptide is aligned with the predicted amino acid sequence of RAD51DΔ9,10 and RAD51DΔ10. RAD51DΔ9,10 contains the first 8 exons of RAD51D followed by four out of frame amino acids (underlined and italicized) encoded by intron 8. RAD51DΔ10 includes amino acids 1–302 of RAD51D followed by five novel amino acids encoded by intronic sequence 5980 bp downstream from the predicted *Rad51d *polyA site. Premature termination codons are indicated by an asterisk, and exon boundaries are represented by a gap in the alignment of the predicted amino acid sequences. (D) RT-PCR for RAD51DΔ9,10 (upper panel) and RAD51DΔ10 (lower panel) from *Mus musculus *tissues with primers Rad51d F3 and Rad51dΔ9,10 R1 or Rad51dΔ10 R1 respectively. Abbreviations: *51D*; RAD51D-FL, Δ3; RAD51DΔ3, Δ5; RAD51DΔ5, Δ9,10; RAD51DΔ9,10, Δ10; RAD51DΔ10.

Mouse *Rad51d *alternative transcripts were originally identified in brain tissue [16]. However, the isolation of corresponding expressed sequence tags in a variety of tissues and cell types suggest that RAD51D isoforms are widely expressed (Table 1). To further investigate, RNA isolated from C57BL/6J mouse tissues were used for RT-PCR expression analysis. A series of overlapping PCRs were designed to detect all *Rad51d *alternative transcripts (Figure 5A). RAD51DΔ3 and RAD51DΔ5 were consistently detected in most tissues examined and their identities verified by restriction enzyme and sequence analysis (not shown). However, RAD51DΔ3 was not detected in ovarian tissue. Additionally, RAD51DΔ5 was not detected in mammary gland and testis, suggesting that these splice isoforms are differentially expressed. Because these same isoforms were identified in human adult and fetal brain cDNA libraries [16], initially named HsTRAD-d1 and HsTRAD-d4 respectively, expression analysis was performed in normal human breast tissue using primers that span from exon 2 to exon 6. Consistent with the results in mouse tissue, 21% of the detectable products corresponded to RAD51DΔ3, whereas RAD51DΔ5 was not detectable above background levels (Figure 5B).

As part of our efforts toward verifying the presence of exons 7 through 10 in *Rad51d *splice variants [15], 3'RACE was performed on total RNA isolated from BALB/c mouse liver tissue. Sequencing of cloned products revealed the existence of two additional alternatively spliced transcripts lacking sequence corresponding to one or both of the final *Rad51d *exons (Figure 5C). Both RAD51DΔ9,10 and RAD51DΔ10 transcripts are derived from the use of alternative splice sites contained within downstream intronic sequence and predicted to result in C-terminal truncated RAD51D isoforms. Notably for RAD51DΔ10, this alternative splice donor site is located 5980 bp downstream, past the predicted *Rad51d *polyadenylation site. In addition, both RAD51DΔ9,10 and RAD51DΔ10 predicted translation products contain a short stretch of novel sequence resulting from the ensuing frameshift. Using unique primers, both RAD51DΔ9,10 and RAD51DΔ10 were demonstrated to be expressed in all tissues examined (Figure 5D).

## Discussion

The studies described here are the first to explore functional properties of RAD51D isoforms predicted to result from alternative splicing [15]. Each splice product was first tested for its ability to interact with RAD51C and XRCC2, proteins known to directly interact with RAD51D and participate as part of the BCDX2 complex [9]. BCDX2 is a complex that binds to DNA nicks, which is one of the key steps during homologous recombination repair of DNA crosslinks and branched structures, which are similar to Holliday junctions [9,33]. Additionally, BCDX2 performs strand-annealing reactions [33]. Previous domain mapping of RAD51 paralog interactions indicated that the amino-terminal portion of RAD51D interacts with XRCC2 and the carboxy-terminal region interacts with RAD51C [29].

The RAD51D+int3 isoform is predicted to contain residues 4–87 of the full-length protein and an additional 20 amino acids from the splice induced frameshift. Consistent with the Miller model [29], this isoform interacted strongly with XRCC2 and failed to interact with RAD51C (Figure 2). However, the RAD51DΔ3 isoform containing the N-terminal domain, but not the linker region, did not interact with XRCC2 but did interact with RAD51C, albeit dependent upon the orientation. These results suggested that residues 60–78 within the RAD51D predicted linker region governs interactions between the XRCC2 and RAD51C paralogs. Therefore, within the CDX2 complex there may be three key interactions: 1) RAD51C with both the N and C-terminal RAD51D domains; 2) interactions of the RAD51D linker region with XRCC; and 3) interactions between the N and C-terminal RAD51D domains via a predicted salt bridge [34]. Electron microscopic analysis showed that RAD51D-XRCC2 alone formed a multimeric ring structure in the absence of DNA, similar to that of RAD52, but formed a filamentous structure unlike that of RAD51/RecA upon addition of ssDNA [35]. Regulating these three types of interactions would likely influence the formation and function of this complex during HR repair.

The RAD51DΔ7b isoform was the only alternative splice product to interact with both RAD51C and XRCC2. Presumably, internal deletions resulting from RAD51DΔ7,8 and RAD51DΔ5 affect integrity of the C-terminal domain, particularly the β-sheet [29]. RAD51DΔ7b is missing a small portion of exon 7, resulting in an in-frame deletion of "GQQRE" between residues 219–223. Because the deletion in RAD51DΔ7b is in proximity to the adenine nucleotide binding sequence of Walker Motif B in RAD51D [25,36], it is possibly required for the hydrolysis of ATP. The ATPase activity of RAD51D is required for its role in the repair of DNA interstrand crosslinks [32,37]. Residues adjacent to Motif B have been found to be critical to ATPase activity in Walker box containing proteins including RAD51D. Notably, conserved glutamate residues in ATP-binding cassette proteins have been proposed to serve as a catalytic base in ATP hydrolysis [38]. Thus, the observation that RAD51DΔ7b did not complement sensitivity of *Rad51d*-deficient cells from DNA interstrand crosslinks caused by mitomycin C is consistent with a role for the "GGQRE" sequence in ATP hydrolysis.

The exclusion of an NLS has been reported or implied from studies of numerous alternative splice products. For example, predicted splice variants of both RAD51C and RAD51B are expected to lack their putative NLS sequences [28,30,31,39]. Given the telomere binding of endogenous RAD51D in human cells [11] and the observation that each of the predicted isoforms of RAD51D includes the "RKIK" putative NLS sequence at the amino terminus, nuclear localization of EGFP-RAD51D isoforms was expected. In contrast, RAD51D was expressed throughout the cell. Another published study, using EGFP-RAD51D, is consistent with this observation [31]. It is conceivable that the presence of the EGFP fusion in the amino-terminal region of RAD51D inhibits access to the "RKIK" sequence whereas the carboxy-terminal NLS present in RAD51C is unaffected. Alternatively, the over-expression of EGFP-RAD51D may saturate the cellular machinery responsible for import. Recently, a RAD51 alternative splice isoform was identified which also lacks exons 9 and 10 [24]. This hRAD51-Δex9 had subtle differences in tissue expression, being primarily expressed in testis. hRAD51-Δex9 was also able to perform strand invasion activity similar to full-length and was localized to the nucleus, likely through an RKR motif introduced by the translational frameshift. In either case, the likelihood remains that the "RKIK" sequence in RAD51D is not recognized as an NLS, and transport of RAD51D into the nucleus occurs as part of a protein complex [31].

The previous protein modeling and interaction studies suggested linker regions were critical for interaction of the RAD51 paralogs. Our results are consistent with this interpretation but also suggest this region may play a role in regulating these interactions. A small region of RAD51C was also identified to be critical for interaction with both RAD51B and XRCC3 [40]. It remains to be determined whether aberrant splicing of *Rad51d *and related HR genes play a role during carcinogenesis. However, two of the human alternative transcripts of *RAD51D *have been identified in tumor derived cells (Table 1) [17], including an EST from a mammary tumor (DN997215) that corresponds to the RAD51DΔ3 isoform. The association of alternative gene products in cancer raises the possibility that the alternative splicing mechanism is a potential target for gene therapy [19,41,42]. Molecular targeting of residues 54–77 in RAD51D could disrupt interactions within the BCDX2 recombinosome and sensitize cells to DNA interstrand crosslinks.

The pattern of protein-protein interactions between isoforms of RAD51D and other RAD51 paralogs described here suggests that RAD51DΔ7b, RAD51DΔ3, and RAD51D+int3 have potential to regulate homologous recombination repair by sequestering members of BCDX2 complex. In addition, three of the alternatively spliced transcripts, RAD51DΔ3, RAD51DΔ8, and RAD51D+int3, harbor premature termination codons and are thus subject to degradation by nonsense-mediated mRNA decay (NMD) [43]. More than one third of all mRNAs derived from alternative splicing are estimated to contain similar nonsense codons [44]. For that reason, it has been proposed that the pre-mRNA splicing and NMD pathways are functionally coupled to provide an additional level of post-transcriptional regulation [45,46]. Therefore, cells could potentially alter gene expression by favoring the splicing of pre-mRNA into an alternative transcript that is targeted to NMD. It is also conceivable that HR may be directed on a tissue selective manner and that upregulation or downregulaton of specific isoforms provides a means of regulation. This hypothesis is supported by the observation that the *Rad51d *alternative transcripts display tissue specific expression patterns (Figure 5). It is also intriguing that RAD51D+int3 corresponds to one of the alternative splice isoforms predicted in humans [16]. During the course of these studies, two new RAD51D isoforms were isolated, RAD51DΔ10 and RAD51DΔ9,10. Further studies are now necessary to explore the two newly discovered variants and determine whether splice isoforms may regulate HR. Finally, it remains to be determined whether these *Rad51d *alternatively spliced products interact with additional known RAD51D interacting proteins BLM and SWS1 [47,48] or contribute to maintenance of telomere integrity.

## Conclusion

Here we report the *Mus musculus Rad51d *alternative transcripts encode predicted proteins capable of making specific interactions with RAD51C and XRCC2. Expression studies revealed the RAD51DΔ3 and RAD51DΔ5 transcripts display tissue specific expression, being detected in each tissue except for mouse ovary or mammary gland and testis respectively. Additionally, we report the identification of two novel, ubiquitously expressed *Mus musculus Rad51d *alternative transcripts. The predicted RAD51D isoforms contain truncated C-terminal ends due to the retention of intron 8 (RAD51DΔ9,10) and the deletion of exon 10 (RAD51DΔ10). The unusual number of alternative splice variants expressed from the *Rad51d *gene compared with the other members of the RAD51 family suggests the RAD51D isoforms potentially regulate specific HR functions.

## Methods

### Plasmid construction

Complementary DNA clones encoding each predicted translation product from alternative splicing of *Mus musculus Rad51d *were generated [15]. The GenBank accession numbers are AB052828.1 (RAD51DΔ8), AB052829.1 (RAD51DΔ7b), AB052830.1 (RAD51DΔ7,8), AB052831.1/AB052832.1 (RAD51DΔ3), AB052833.1 (RAD51DΔ5), and AB052834.1 (RAD51D+int3).

RAD51DΔ8 was generated by ligating a 734 bp sequence resulting from a NheI/StuI digest of full-length *Rad51d *to a dsDNA made by annealing Rad51dΔ8StuI and Rad51dΔ8BamHI oligos. RAD51DΔ7b was produced by PCR based site directed mutagenesis using primers Rad51dΔ7b F1and Rad51dΔ7b R1. The RAD51DΔ7,8 expression construct was generated by multiple subcloning steps. First, full-length *Rad51d *was digested with XhoI/BamHI, and the resulting 248 bp fragment cloned into pDsRed2-N1 (Clontech, Palo Alto, CA) to generate the RAD51DΔ7,8 3' end. Second, a 622 bp fragment resulting from NheI/AvaII digest of full-length *Rad51d *was isolated and subcloned into the NheI/AvaII sites of the RAD51DΔ7,8 3' end construct. Finally, duplexed oligos of Rad51dΔ7,8AvaII and Rad51dΔ7,8XhoI were subcloned into the pDsRed2-N1 construct generated above prior to the final cloning step. RAD51DΔ3 was constructed by ligating the 200 bp fragment resulting from a NheI/BstXI digest of full-length *Rad51d *to duplexed oligos Rad51dΔ3BstXI. The 5' end of RAD51DΔ5 was amplified using Rad51dKpnIF and Rad51dΔ5NheBamR and cloned into pUC19. The 3' end was amplified using Rad51dΔ5NheF and Rad51dBamR primers prior to subcloning into the RAD51DΔ5 5' pUC19 construct. RAD51D+int3 was generated by a restriction digest of full-length *Rad51d *using NheI and BsrFI enzymes. The resulting 323 bp fragment was ligated with duplexed oligos Rad51d+int3F and Rad51d+int3R to construct the final insert. The make-up of each splice product cDNA was confirmed by double-strand sequencing and cloned into the NheI/BamHI sites of the pcDNA3.1/Hygro (+) vector (Invitrogen, Carlsbad, CA) with the exception of RAD51DΔ5, which was cloned into the KpnI/BamHI sites. Each construct contains an influenza HA epitope-tagging sequence at the 5' end.

Full-length *Mus musculus Rad51c *was obtained by reverse transcribing total RNA from mouse kidney as described above. The DNA was PCR amplified using Rad51cKpnF and Rad51cBamR primers. Full-length *Mus musculus Xrcc2 *was amplified from IMAGE clone 5357630 (American Type Culture Collection; Manassas, VA) using XRCC2F and XRCC2R primers. *Rad51c *and *Xrcc2 *amplification products were digested with KpnI/BamHI and subcloned into the pcDNA3.1/Hyro(+) vector encoding an HA epitope tagging sequence at the 5' end. The RAD51D (4–77) and (77–329) deletion constructs were a generous gift of Dr. Joanna Albala (University of California, Davis, Sacramento, CA).

### Yeast two-hybrid analysis

*MmRad51d*, *MmRad51c*, and *MmXrcc2 *inserts containing the N-terminal hemaglutinin tag were cloned into the EcoRI/BamHI restriction sites of pGADT7 and pGBKT7 yeast two-hybrid vectors encoding the activating and DNA binding domains of GAL4 respectively (Clontech). Haploid transformants of AH109 and Y187 were generated using the EZ Transformation Kit II (Zymo, Orange, CA). Matings were performed on YPDA plates 16–24 hours prior to replica plating on dropout media. The presence of diploids was confirmed by growth on -Leu/-Trp medium following 3 days of incubation at 30°C. Mated strains containing interacting proteins were subsequently analyzed for growth on -Ade/-Leu/-His/-Trp medium. Images were captured following 10 days of growth at 30°C. Colony lift assays were performed according to the yeast protocols handbook (PT3024-1, Clontech). To determine the strength of protein-protein interactions, liquid β-galactosidase assays were performed using ortho-nitrophenyl-β-galactopyranoside as a substrate (ONPG; Sigma, St. Louis, MO) essentially as described [15]. All β-galactosidase assays were performed in triplicate with constructs in both the GAL4 activating and DNA binding domains.

### Localization studies

To generate green fluorescent protein fusions of the alternative splice variants, each was cloned into the KpnI/BamHI sites of a pEGFP-C1 based vector (BD Biosciences). Constructs were transiently expressed in *Rad51d*-deficient MEFs grown on glass coverslips as described [6]. Cells were harvested 24 hours post-transfection and coverslips were washed in 1× PBS prior to fixation with 4% paraformaldehyde for 10 minutes at room temperature. Fixed cells were subsequently washed and permeablized with 0.3% Triton-X 100 for 5 minutes. Nuclei were counterstained with 0.2 μg/mL of 4',6-diamidino-2-phenylindole dihydrochloride hydrate (Sigma) in 1× PBS for 10 minutes and coded before mounting on glass slides with fluorescent mounting medium (DakoCytomation, Carpinteria, CA). Slides were viewed using a Nikon Eclipse fluorescent microscope, and images captured using a 60× dry lens objective.

### Complementation analysis

*Rad51d*^-/-^*Trp53*^-/- ^MEFs were grown in monolayer culture as described [6]. One microgram of each plasmid construct was transfected into *Rad51d*^-/- ^*Trp53*^-/- ^MEFs using Lipofectamine with Plus reagent in six-well format according to the manufacturer's instructions (Invitrogen). Twenty-four hours post-transfection cells were trypsinized, mixed, and divided equally (~7.5 × 10^5 ^cells per dish) onto two 150 mm dishes. Twenty-four hours after plating, cells were selected in growth medium containing 200 μg/mL Hygromycin B with or without the addition of mitomycin C (4 ng/mL). Colonies were harvested approximately twelve days after selection and fixed with 100% ice-cold methanol prior to staining with Giemsa. Colonies containing ≥ 50 cells were scored positive. The percentage of mitomycin C resistant colonies was determined by dividing the number surviving selection with mitomycin C and Hygromycin B by the number that grew in the presence of Hygromycin B alone on the duplicate plate. Statistical significance of the experimental data was determined using SPSS^® ^version 11.0 for Mac OS X. The mean numbers of percentage mitomycin C resistance for each construct were compared by ANOVA.

### Protein expression and western blotting

Protein expression and Western blotting was conducted as described previously [32] with the exception that each sample was resolved on a 15% SDS-PAGE. For detection of the smaller RAD51DΔ3 and RAD51D+int3 constructs, cells harvested from two separate wells per sample were pooled and 200 μg of each sample was resolved on 10–20% Tricine gel followed by transfer onto a 0.1 micron nitrocellulose membrane (Protran BA-79, Whattman).

### Expression analysis of Rad51d alternative splice variants

Total RNA was purified from C57BL/6J mouse tissues (Aurum Total RNA Isolation kit; Bio-Rad) and reverse transcribed using both oligo(dT) and random hexamers (iScript cDNA Synthesis kit; Bio-Rad). Primers specific for *Rad51d *(Rad51d F1, Rad51d F2, Rad51d R1, and Rad51d R2), *Rad51dΔ9,10 *(Rad51d F3 and Rad51dΔ9,10 R1), or *Rad51dΔ10 *(Rad51d F7 and Rad51dΔ10 R1) were employed. Amplification was performed under the following conditions: 94°C for 3 min, followed by 35 cycles at 94°C for 30 s, 60°C for 30 s, and 72°C for 1 min. All sequences of primers used in this study are listed in Table 2. For expression analysis in human cells, total RNA from normal breast tissue (Stratagene, #735044) was reverse transcribed as above and PCR amplified for 35 cycles using the HSTRF1 and HSTRR1 primers, corresponding to exon 2 and exon 6 respectively, as initially described [16]. The products were separated on a 5% polyacrylamide gel. Image J software  was used for quantitative analysis of band intensity levels by the gel analysis function.

**Table 2:** Primers used for cloning and expression studies

Primer Name	Sequence
Rad51d F1	5'-GCTGACTTGGAGGAAGTAGCCCAGAAGTGT-3'
Rad51d F2	5'-CTACTTGATGCTGGCCTCTATACTGG-3'
Rad51d F3	5'-GCAGGAAGCAACTTCTTCAGGCG-3'
Rad51d R1	5'-AGCCTGTAGTAGCTGGAGGAGG-3'
Rad51d R2	5'-TGAACGCACCACCTGTATCCTCTGGAGAG-3'
Rad51dΔ9,10 R1	5'-GTTCTAAGACAGACAGAGCAC-3'
Rad51d Δ10 R1	5'-GAGACACAGGTTCTTCACCACAC-3'
Rad51dΔ8StuI	5'-TGACCAACCACTTGACTCGAGATTGGGATGGTAGG-3'
Rad51dΔ8BamHI	5'-GATCCCTACCATCCCAATCTCGAGTCAAGTGGTTGGTCA-3'
Rad51dΔ7b F1	5'-GCCCCACTTCTGGGAGGCCTGGCCTTGATG-3'
Rad51DΔ7b R1	5'-CATCAAGGCCAGGCCTCCCAGAAGTGGGGC-3'
Rad51dΔ7,8AvaII	5'-GACCTTCGCGGCACCATAGCCCAGCAGGTGACCAACCACTTGAC-3'
Rad51dΔ7,8XhoI	5'-TGCAGTCAAGTGGTTGGTCACCTGCTGGGCTATGGTGCCGCGAAG-3'
Rad51dΔ3BstXI	5'-GTGGCTTGTCCTACAAGCCTGGACAAACTACTTGAG-3'
Rad51dKpnIF	5'-GGACTATGGGTACCCTCAGGGCA-3'
Rad51dΔ5NheBamR	5'-CAGGATCCAGCTAGCCTGGGTTTTGCC-3'
Rad51dΔ5NheF	5'-AACTCAGGCTAGCGCTCTCCAGAGG-3'
Rad51dBamR	5'-CAGTGGATCCCAATCAACAGTGTCA-3'
Rad51d+int3F	5'-CCGGCATCGGAAGGTTTGTATGCGAACCTGGACAAGCCTGGACAA ACTACTTGATGCTGGCCTCTATACTGGGGAAGTGAG-3'
Rad51d+int3R	5'-GATCCCTCACTTCCCCAGTATAGAGGCCAGCATCAAGTAGTTTGTC CAGGCTTGTCCAGGTTCGCATACAAACCTTCCGATG-3'
Rad51dex6F	5'-CCAGAGGATACAGGTGGTGCGTTCATTTGAC-3'
Rad51dΔ9,10BamR	5'-CCGGGATCCGTTCTAAGACAGACAGAGCAC-3'
Rad51dΔ10BamR	5'-CCGGGATCCGAGCACAGGTTCTTCACCA-3'
Rad51cKpnF	5'-CTTGGTACCCAGCGGGAGTTGGTGGGT-3'
Rad51cBamR	5'-CAGTTAACTGGATCCACTGGCA-3'
XRCC2F	5'-ATGCTACGGCTCGTGACAGTTCTT-3'
XRCC2R	5'-AGAAGATGACCCTGTGCTTCACGA-3'

### Rapid amplification of Rad51d 3' cDNA ends

Total RNA from pooled adult mouse BALB/c livers (Clontech) was used for 3'RACE according to manufacturer instructions (Gene Racer kit; Invitrogen). Amplification was performed using the GeneRacer 3' Primer and Rad51d F1. Nested PCR was carried out with the Gene Racer Nested Primer and Rad51d F2. Amplification products were cloned into pCR4-TOPO (Invitrogen), and sequences of newly identified isoforms deposited into GenBank [GenBank: EU627687 (RAD51DΔ9,10), EU627688 (RAD51DΔ10)].

## Authors' contributions

AMG and BDY equally contributed to the work presented in this manuscript. AMG and DLP conceived this study, and AMG was responsible for generating the cDNA clones, complementation studies, Y2H and localization analyses. BDY identified the new splice isoforms and performed the transcription studies. CR was responsible for verification of transgene expression. CM performed the molecular modeling. DLP participated as a supervisor in study design and analysis. He is responsible for manuscript drafts, along with AMG and BDY, and has given final approval for the version to be published.

## Supplementary Material

Additional file 1 **Intracellular localization of RAD51D isoforms.** (A) Localization of over-expressed EGFP-RAD51D isoforms. Panels represent images taken from cells transfected with the following DNA constructs: EGFP vector control (a), EGFP-RAD51C (b), EGFP-RAD51D-FL (c), EGFP-RAD51DΔ8 (d), EGFP-RAD51DΔ7b (e), EGFP-RAD51DΔ7,8 (f), EGFP-RAD51DΔ3 (g), EGFP-RAD51DΔ5 (h), EGFP-RAD51D+int3 (i). (B) Repair activity of RAD51D tagged with amino-terminal enhanced green fluorescent protein. *Rad51d*-deficient mouse embryonic fibroblasts were challenged with 4 ng/mL mitomycin C following transfection. Error bars represent the standard error. Abbreviations: *51D*; RAD51D-FL (no tag), *GFP*; EGFP-RAD51D-FL, *Vec*; pcDNA3.1/Hygro vector. Click here for file

## References

[B1] Li X, Heyer WD (2008). Homologous recombination in DNA repair and DNA damage tolerance. Cell Res.

[B2] Thacker J (2005). The RAD51 gene family, genetic instability and cancer. Cancer Lett.

[B3] West SC (2003). Molecular views of recombination proteins and their control. Nat Rev Mol Cell Biol.

[B4] Takata M, Sasaki MS, Sonoda E, Fukushima T, Morrison C, Albala JS, Swagemakers SM, Kanaar R, Thompson LH, Takeda S (2000). The Rad51 paralog Rad51B promotes homologous recombinational repair. Mol Cell Biol.

[B5] French CA, Masson JY, Griffin CS, O'Regan P, West SC, Thacker J (2002). Role of mammalian RAD51L2 (RAD51C) in recombination and genetic stability. J Biol Chem.

[B6] Smiraldo PG, Gruver AM, Osborn JC, Pittman DL (2005). Extensive chromosomal instability in Rad51d-deficient mouse cells. Cancer Res.

[B7] Liu N, Lamerdin JE, Tebbs RS, Schild D, Tucker JD, Shen MR, Brookman KW, Siciliano MJ, Walter CA, Fan W, Narayana LS, Zhou ZQ, Adamson AW, Sorensen KJ, Chen DJ, Jones NJ, Thompson LH (1998). XRCC2 and XRCC3, new human Rad51-family members, promote chromosome stability and protect against DNA cross-links and other damages. Mol Cell.

[B8] Yoshida K, Kondoh G, Matsuda Y, Habu T, Nishimune Y, Morita T (1998). The mouse RecA-like gene Dmc1 is required for homologous chromosome synapsis during meiosis. Mol Cell.

[B9] Masson JY, Tarsounas MC, Stasiak AZ, Stasiak A, Shah R, McIlwraith MJ, Benson FE, West SC (2001). Identification and purification of two distinct complexes containing the five RAD51 paralogs. Genes Dev.

[B10] Wiese C, Collins DW, Albala JS, Thompson LH, Kronenberg A, Schild D (2002). Interactions involving the Rad51 paralogs Rad51C and XRCC3 in human cells. Nucleic Acids Res.

[B11] Tarsounas M, Munoz P, Claas A, Smiraldo PG, Pittman DL, Blasco MA, West SC (2004). Telomere maintenance requires the RAD51D recombination/repair protein. Cell.

[B12] Birzele F, Csaba G, Zimmer R (2008). Alternative splicing and protein structure evolution. Nucleic Acids Res.

[B13] Ben-Dov C, Hartmann B, Lundgren J, Valcarcel J (2008). Genome-wide analysis of alternative pre-mRNA splicing. J Biol Chem.

[B14] Romero PR, Zaidi S, Fang YY, Uversky VN, Radivojac P, Oldfield CJ, Cortese MS, Sickmeier M, LeGall T, Obradovic Z, Dunker AK (2006). Alternative splicing in concert with protein intrinsic disorder enables increased functional diversity in multicellular organisms. Proc Natl Acad Sci USA.

[B15] Kawabata M, Akiyama K, Kawabata T (2004). Genomic structure and multiple alternative transcripts of the mouse TRAD/RAD51L3/RAD51D gene, a member of the recA/RAD51 gene family. Biochim Biophys Acta.

[B16] Kawabata M, Saeki K (1999). Multiple alternative transcripts of the human homologue of the mouse TRAD/R51H3/RAD51D gene, a member of the rec A/RAD51 gene family. Biochem Biophys Res Commun.

[B17] Pospisil H, Herrmann A, Bortfeldt RH, Reich JG (2004). EASED: Extended Alternatively Spliced EST Database. Nucleic Acids Res.

[B18] Durrant WE, Wang S, Dong X (2007). Arabidopsis SNI1 and RAD51D regulate both gene transcription and DNA recombination during the defense response. Proc Natl Acad Sci USA.

[B19] Venables JP (2004). Aberrant and alternative splicing in cancer. Cancer Res.

[B20] Srebrow A, Kornblihtt AR (2006). The connection between splicing and cancer. J Cell Sci.

[B21] Thorpe PH, Marrero VA, Savitzky MH, Sunjevaric I, Freeman TC, Rothstein R (2006). Cells expressing murine RAD52 splice variants favor sister chromatid repair. Mol Cell Biol.

[B22] Farrugia DJ, Agarwal MK, Pankratz VS, Deffenbaugh AM, Pruss D, Frye C, Wadum L, Johnson K, Mentlick J, Tavtigian SV, Goldgar DE, Couch FJ (2008). Functional assays for classification of BRCA2 variants of uncertain significance. Cancer Res.

[B23] Claes K, Poppe B, Machackova E, Coene I, Foretova L, De Paepe A, Messiaen L (2003). Differentiating pathogenic mutations from polymorphic alterations in the splice sites of BRCA1 and BRCA2. Genes Chromosomes Cancer.

[B24] Park JY, Yoo HW, Kim BR, Park R, Choi SY, Kim Y (2008). Identification of a novel human Rad51 variant that promotes DNA strand exchange. Nucleic Acids Res.

[B25] Pittman DL, Weinberg LR, Schimenti JC (1998). Identification, characterization, and genetic mapping of Rad51d, a new mouse and human RAD51/RecA-related gene. Genomics.

[B26] Thanaraj TA, Stamm S, Clark F, Riethoven JJ, Le Texier V, Muilu J (2004). ASD: the Alternative Splicing Database. Nucleic Acids Res.

[B27] Schild D, Lio YC, Collins DW, Tsomondo T, Chen DJ (2000). Evidence for simultaneous protein interactions between human Rad51 paralogs. J Biol Chem.

[B28] Dosanjh MK, Collins DW, Fan W, Lennon GG, Albala JS, Shen Z, Schild D (1998). Isolation and characterization of RAD51C, a new human member of the RAD51 family of related genes. Nucleic Acids Res.

[B29] Miller KA, Sawicka D, Barsky D, Albala JS (2004). Domain mapping of the Rad51 paralog protein complexes. Nucleic Acids Res.

[B30] French CA, Tambini CE, Thacker J (2003). Identification of functional domains in the RAD51L2 (RAD51C) protein and its requirement for gene conversion. J Biol Chem.

[B31] Miller KA, Hinz JM, Yamada NA, Thompson LH, Albala JS (2005). Nuclear localization of Rad51B is independent of Rad51C and BRCA2. Mutagenesis.

[B32] Gruver AM, Miller KA, Rajesh C, Smiraldo PG, Kaliyaperumal S, Balder R, Stiles KM, Albala JS, Pittman DL (2005). The ATPase motif in RAD51D is required for resistance to DNA interstrand crosslinking agents and interaction with RAD51C. Mutagenesis.

[B33] Yokoyama H, Sarai N, Kagawa W, Enomoto R, Shibata T, Kurumizaka H, Yokoyama S (2004). Preferential binding to branched DNA strands and strand-annealing activity of the human Rad51B, Rad51C, Rad51D and Xrcc2 protein complex. Nucleic Acids Res.

[B34] Rodriguez-Lopez R, Osorio A, Ribas G, Pollan M, Sanchez-Pulido L, de la Hoya M, Ruibal A, Zamora P, Arias JI, Salazar R, Vega A, Martinez JI, Esteban-Cardenosa E, Alonso C, Leton R, Urioste Azcorra M, Miner C, Armengod ME, Carracedo A, Gonzalez-Sarmiento R, Caldes T, Diez O, Benitez J (2004). The variant E233G of the RAD51D gene could be a low-penetrance allele in high-risk breast cancer families without BRCA1/2 mutations. Int J Cancer.

[B35] Kurumizaka H, Ikawa S, Nakada M, Enomoto R, Kagawa W, Kinebuchi T, Yamazoe M, Yokoyama S, Shibata T (2002). Homologous pairing and ring and filament structure formation activities of the human Xrcc2*Rad51D complex. J Biol Chem.

[B36] Pittman DL, Schimenti JC (2000). Midgestation lethality in mice deficient for the RecA-related gene, Rad51d/Rad51l3. Genesis.

[B37] Wiese C, Hinz JM, Tebbs RS, Nham PB, Urbin SS, Collins DW, Thompson LH, Schild D (2006). Disparate requirements for the Walker A and B ATPase motifs of human RAD51D in homologous recombination. Nucleic Acids Res.

[B38] Orelle C, Dalmas O, Gros P, Di Pietro A, Jault JM (2003). The conserved glutamate residue adjacent to the Walker-B motif is the catalytic base for ATP hydrolysis in the ATP-binding cassette transporter BmrA. J Biol Chem.

[B39] Schoenmakers EF, Huysmans C, Ven WJ Van de (1999). Allelic knockout of novel splice variants of human recombination repair gene RAD51B in t(12;14) uterine leiomyomas. Cancer Res.

[B40] Connell PP, Siddiqui N, Hoffman S, Kuang A, Khatipov EA, Weichselbaum RR, Bishop DK (2004). A hot spot for RAD51C interactions revealed by a peptide that sensitizes cells to cisplatin. Cancer Res.

[B41] Sampath J, Pelus LM (2007). Alternative splice variants of survivin as potential targets in cancer. Curr Drug Discov Technol.

[B42] Gaur RK (2006). RNA interference: a potential therapeutic tool for silencing splice isoforms linked to human diseases. Biotechniques.

[B43] Chang YF, Imam JS, Wilkinson MF (2007). The nonsense-mediated decay RNA surveillance pathway. Annu Rev Biochem.

[B44] Lewis BP, Green RE, Brenner SE (2003). Evidence for the widespread coupling of alternative splicing and nonsense-mediated mRNA decay in humans. Proc Natl Acad Sci USA.

[B45] Lareau LF, Brooks AN, Soergel DA, Meng Q, Brenner SE (2007). The coupling of alternative splicing and nonsense-mediated mRNA decay. Adv Exp Med Biol.

[B46] Lejeune F, Maquat LE (2005). Mechanistic links between nonsense-mediated mRNA decay and pre-mRNA splicing in mammalian cells. Curr Opin Cell Biol.

[B47] Braybrooke JP, Li JL, Wu L, Caple F, Benson FE, Hickson ID (2003). Functional interaction between the Bloom's syndrome helicase and the RAD51 paralog, RAD51L3 (RAD51D). J Biol Chem.

[B48] Martin V, Chahwan C, Gao H, Blais V, Wohlschlegel J, Yates JRr, McGowan CH, Russell P (2006). Sws1 is a conserved regulator of homologous recombination in eukaryotic cells. EMBO J.

