# Supplementary figures and images for: Functional characterization and identification of mouse Rad51d splice variants
Source: BMC Mol Biol. 2009 Mar 27;10:27. doi: 10.1186/1471-2199-10-27 (PMC2667185; doi:10.1186/1471-2199-10-27)

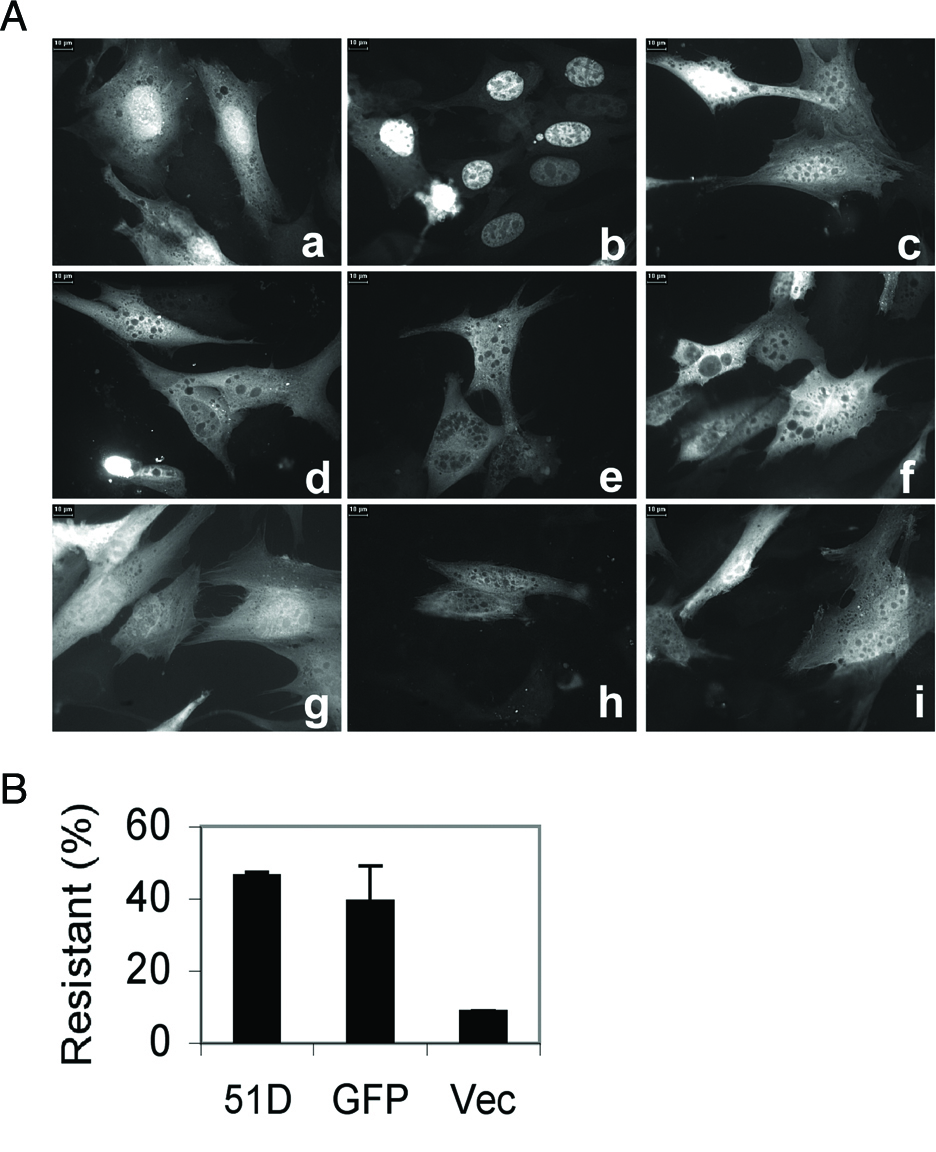

Supplement: Additional file 1 — Intracellular localization of RAD51D isoforms. (A) Localization of over-expressed EGFP-RAD51D isoforms. Panels represent images taken from cells transfected with the following DNA constructs: EGFP vector control (a), EGFP-RAD51C (b), EGFP-RAD51D-FL (c), EGFP-RAD51DΔ8 (d), EGFP-RAD51DΔ7b (e), EGFP-RAD51DΔ7,8 (f), EGFP-RAD51DΔ3 (g), EGFP-RAD51DΔ5 (h), EGFP-RAD51D+int3 (i). (B) Repair activity of RAD51D tagged with amino-terminal enhanced green fluorescent protein. Rad51d-deficient mouse embryonic fibroblasts were challenged with 4 ng/mL mitomycin C following transfection. Error bars represent the standard error. Abbreviations: 51D; RAD51D-FL (no tag), GFP; EGFP-RAD51D-FL, Vec; pcDNA3.1/Hygro vector. [file 1471-2199-10-27-S1.jpeg]
